# Endoscopic resection of solitary fibrous tumor of the ethmoid: Case report review of the literature

**DOI:** 10.1016/j.amsu.2020.11.073

**Published:** 2020-12-07

**Authors:** Chaker Kaoutar, Ahmed Brahim Ahmedou, Berrada Omar, Bouzbouz Anas, Youssef Oukessou, Redallah Abada, Rouadi Sami, Roubal Mohamed, Mahtar Mohamed, Regragui Meriem, Karkouri Mehdi

**Affiliations:** a ENT Department, Face and Neck Surgery, Hospital August, 20’1953, University Hospital Centre IBN ROCHD, Casablanca, Kingdom of Morocco; b Pathology Department, Centre IBN ROCHD, Casablanca, Kingdom of Morocco

**Keywords:** olitary fibrous tumour, Sinonasal tract, Endoscopic approach

## Abstract

**Introduction:**

Solitary fibrous tumour (SFT), as are benign neoplasms of fibroblastic cells. Nasosinusal localisation is exremely rare, difficult to diagnose and to manage.

**Case report:**

We report a rare case of Solitary fibrous tumour in the nasal cavity in a 47-year-old- woman, with complete surgical resection.

**Discussion:**

SFTs are of mesenchymal origin, mainly from serous membranes. The head and neck region is affected with a percentage ranging from 5 to 27%. On the other hand, LTS unusually affects the nasal tract (NTS). Because of this rarity and its variable morphological appearance, it is difficult to distinguish TNS from other mesenchymal lesions.

**Conclusion:**

Although there are no standard clinical guidelines, the preferred treatment for FLS is radical surgical resection.

## Highlights

• Solitary fibrous tumour is an uncommon fusion neoplasm of spindled fibroblastic cells set in a branching vasculature with an unclear biologic behavior.
• Nasosinusal localisation is exremely rare, difficult to diagnose and to manage.
• Complete resecability is the most important prognostic factor in the treatment of SFT.
• Advantages of the endoscopic approach include a panoramic and large view without external incision, low postoperative morbidity and a shorter hospital stay.

## 1 Introduction

Solitary fibrous tumour (SFT) or submesothelial fibroma or benign fibrous mesothelioma [1], is an uncommon fusion neoplasm of spindled fibroblastic cells set in a branching vasculature with an unclear biologic behavior. SFTs are generally benign neoplasms and only 10–15% are malignant [2]. SFT have mesenchymal origin which explains why it arises mostly from serous membranes [3]. The head and neck region is affected with a pourcentage of ranges from 5 to 27% [4]. It affects preferentially the oral cavity and orbit [5,6] A. In contrast, SFT unusualy affects the sinonasal tract (SNT). A limited number of SNT SFTs have been reported, in english literature, most of them are case reports or small series [5]. Owing to this rarity and its variable morphologic appearance, it is hard to distinguish SFT from other mesenchymal lesions [7]. Even though no standard clinical treatment guidelines, the preferred treatment for SFT is radical surgical resection [8]. We present a case of SFT of the ethmoid in accordance with SCARE criteria [9], to raise the attention of surgeons to this type of tumor and endoscopic management .No recurrence was seen in long-term follow-up.

## 2 Case report

47 years old woman with no history of tobacco use, or exposure to wood dust or other known carcinogens, reported 2 years history of permanently left nasal obstruction. She has reported anosmia and intermittent epistaxis for the past six months with hemicranial headaches. Ophthalmological examination was normal. His medical history and family history are otherwise unremarkable.

**Fig. 1:**
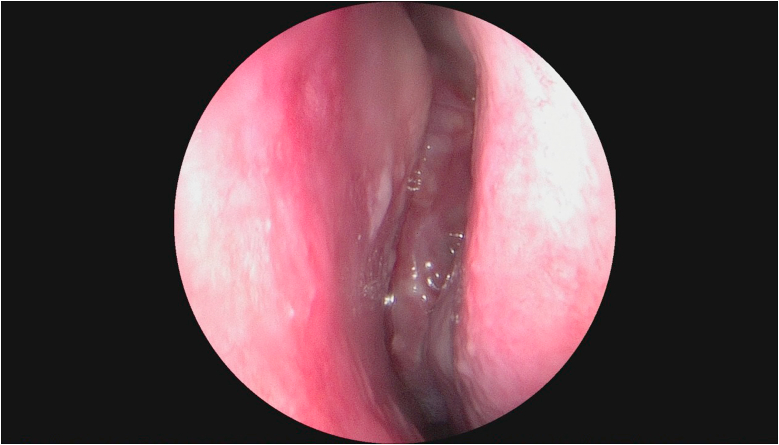
The endoscopic aspect showed a lobulated mass, above of left medial turbinate.

**Fig. 2:**
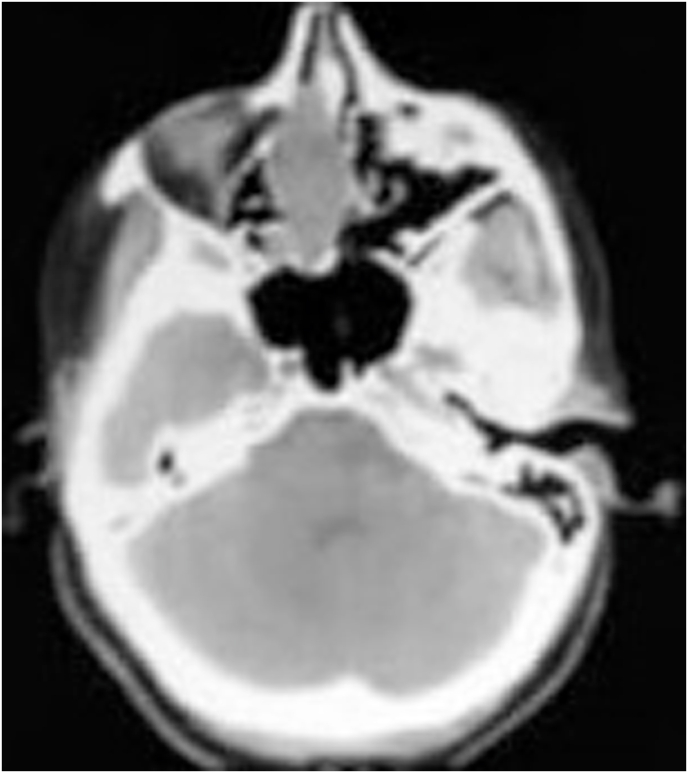
(CT) scan demonstrated an expansive process occupying the ethmoidal cells.

Examination of the left nasal cavity found a lobulated mass bleeding on contact, above of left medial turbinate (Fig. 1), the right nasal cavity and the remainder of the physical examination were unremarkable. Computed tomographic scan revealed an expansive process occupying the ethmoidal cells (Fig. 2).

**Fig. 3:**
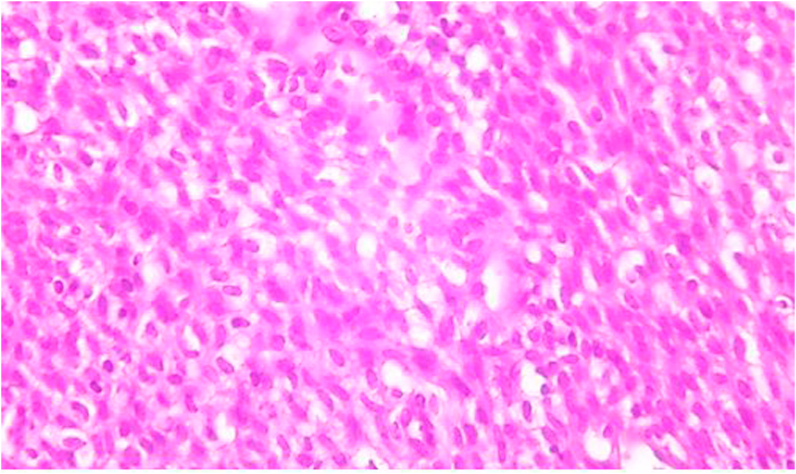
aspect showing a spindle cell with minimal pleomorphism, no atypia, no mitosis.

To better assess the extent and origin of the mass, a MRI with injection of gadolinium was performed, showing a tumor process of the ethmoidal cells, intensely enhanced after injection.The histopathological analysis showed a spindle cell with minimal pleomorphism, no atypia, no mitosis (Fig. 3).

**Fig. 4:**
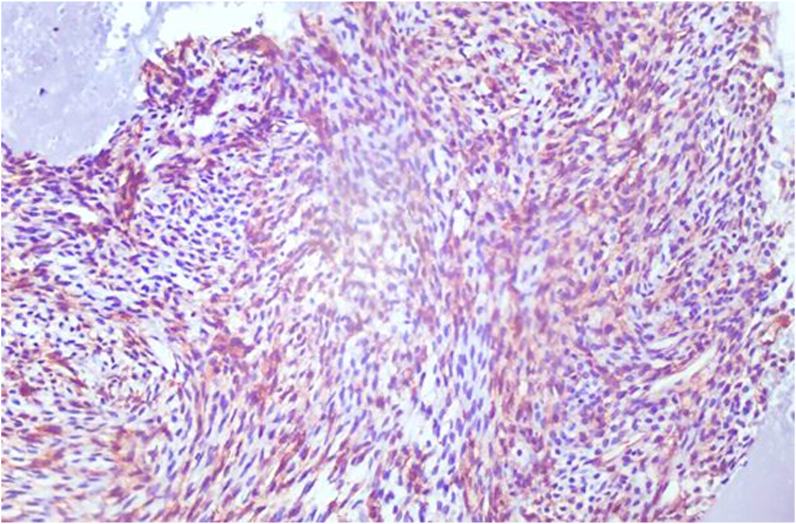
a diffuse expression of CD34 on histologic examination.

**Fig. 5:**
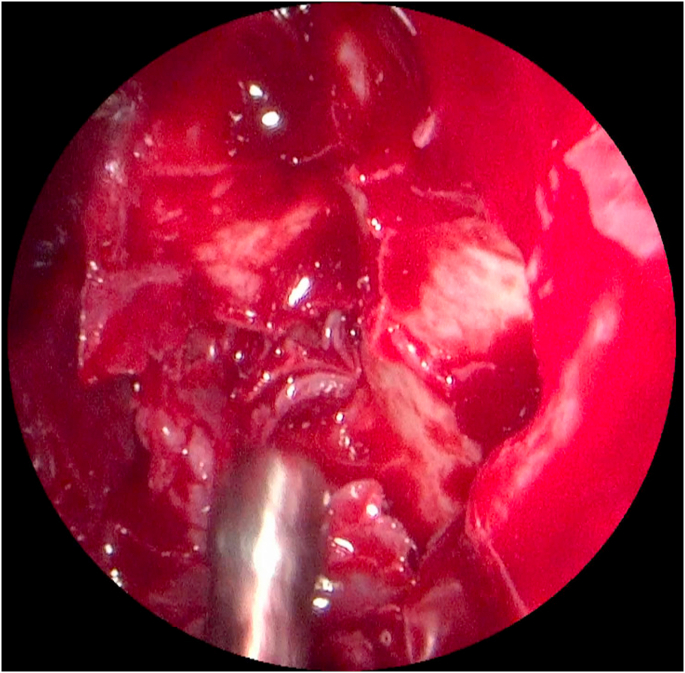
Endoscopic resection with ethmoidectomy.

An immunohistochemical study has been carried out, it shows a diffuse expression of CD34 (Fig. 4). Exclusive endoscopic resection was performed, and the tumor was completely excised. The ethmoidectomy is done. (Fig. 5).

## 3 Discussion

The first description of SFT was in 1931 by Klemperer and Rabin [10]. It is a rare soft tissue spindle cell tumor that may occur in every site of the body, but arises commonly from pleura and and peritoneum. It rarely involve the nasal or the paranasal cavities. Most of the time, SFT are benign neoplasms and malignancy represent only 10–15% [2].

Generally occur in adults in their third to fourth decade of life; with extemes ranging from 9 to 86 years with no significant difference between males and females [11]. Clinically, SFTs present themselves as a slow-growing asymptomatic mass; if symptomatic most of patients presented obstruction and/or epistaxis, evolving for an average of 18.5 months. Just a minority of patients reported symptoms of orbital involvement by the tumor such as proptosis, epiphora or visual field changes [12,13]. The majorities of tumors were unilateral with a mean size of 4.7 cm, and involved the nasal cavity alone followed by combined involvement of the nasal cavity and paranasal sinuses. Usually these tumors are unilateral with a mean size of 4.7 cm with extremes ranging from 2.8 to 8cm in its major axis [14]. Macroscopically, several authors described SFTs as pedicled or sessile, reddish, pinkish, white, oval, circular in shape, well encapsulated fibrous masses with rich vascularization [15]. CT findings are represented essentially by a smooth expansile soft tissue mass, usually well-encapsulated, with bone remodeling, local absorption or erosion from pressure effect. SFT are showed as homogenous isoattenuation on non-contrast CT, thus marked enhancement appears after the administration of contrast material. IMR findings include: well-circumscibed solid mass with hypo- to isointense on T1-weighted images associate to a prominent and heterogeneous enhancement after administration of gadolinuim. Ofenly hyperintensity on T2- weighted images can be seen [16] Inverted papilloma, hemangioma, angiomatous polyps, hemangiopericytoma and juvenile angiofibroma are the main differential diagnosis of sinonasal SFT [15].

Histopathologically, the important feature of this tumor is it composition of bland spindle cells set in a collagenous stroma with typical variation in cellularity with both hypo- and hyper-cellular areas are objectified. The presence of areas of hyalinization adjacent to collagen deposits is also a characteristic of SFT. Another characteristic feature is the presence of a prominent vascular network with dilated, stellate, branching or staghorn shaped vessels. Mature adipose tissue within SFT and pseudovascular spaces lined with multinucleated stromal giant cells are histologic variant [5,16]. Typical features of malignats SFTs, microscopically are defined by the presence of nuclear atypia, necrosis,increased cellularity, more than 4 mitoses per 10 HPF [17]. Immunohistochemically and generally, CD 34, vimentin, bcl-2 and CD 99 positive and S-100 negative [18].The expression of CD 34 CD34 and Vimentin is a typical finding yet, it is not entirely specific for SFT; it can be showed in variety of spindle cell neoplasms like dermatofibrosarcoma protuberans or neural tumors [19]. Recently, STAT6 has became consistently a reliable specific marker for the diagnosis of SFT irrespective of anatomic site. Nuclear expression of STAT6 protein is believed to reflect the presence of a NAB2–STAT6 gene fusion that characterises these tumors which is resulting from a paracentric inversion in chromosome 12q13 [20]. The sensitivity of STAT6 for a diagnosis of SFT overshoots 95% when it is associated to strong and diffuse nuclear expression [4,5,21,22]. The specificity of the nuclear expression of STAT6 is due to the fact that only 2% non SFT mesenchymal neoplasms express both nuclear and cytoplasmic positivity [22], compaired to just nuclear reactivity seen in SFT. Although radiotherapy and chemotherapy have been used, surgery is the treatment of choice for SFTs. Authors believe that the complete resectability is the most important prognostic factor in the treatment of SFTs [23], because all of the published cases of nasal SFTs were treated with surgical resection regardless of an adjuvant radiation or embolisation had been associated or not [24]. Among the advantages of endoscopic approach permits a wide and panoramic view and magnification with no external incision, low postoperative morbidity, and shorter hospital stay [25]. However is often difficult to assess margins when endoscopic surgical approaches are frequently used in this anatomic region. Thus, adequate excision is often left to be determined by the surgeon. Although radiation and chemotherapy have been used to manage locally advanced or recurrent disease, additional therapy beyond surgical resection is not required for the majority of SNT SFTs [7,26].The prognosis of SFTs in SNT is considered favorable even though these tumors show variable biologic behavior. Among all the reported cases of SNT SFT only five patients developed local recurrence after resection were no instances of metastasis or death had been showed. Five patients developed local recurrence at 3–69 months after resection [27]. As such, patients need continued follow-up and assessment.

## 4 Conclusion

SFT is a rare soft tissue spindle cell tumour that can occur in any area of the body. It rarely involves the nasal or paranasal cavities. Most often FTS are benign neoplasms. Complete resecability is the most important prognostic factor in the treatment of SFT. Advantages of the endoscopic approach include a panoramic and large view without external incision, low postoperative morbidity and a shorter hospital stay.

## Ethical approval

I declare on my honor that the ethical approval has been exempted by my establishment.

## Funding

None.

## Author contribution

Chaker Kaoutar: Corresponding author writing the paper. Ahmed Brahim Ahmedou: writing the paper. Youssef Oukessou: study concept. Sami Rouadi:study concept. Reda Abada: study concept. Mohammed Roubal :correction of the paper.

## Registration of research studies

Researchregistry5198.

## Guarantor

DR AHMED BRAHIM AHMEDOU.

## Consent

Written informed consent was obtained from the patient for publication of this case report and accompanying images.

## Provenance and peer review

Not commissioned, externally peer reviewed.

## Declaration of competing interest

The authors declare having no conflicts of interest for this article.
